# Whole-genome re-sequencing association study for direct genetic effects and social genetic effects of six growth traits in Large White pigs

**DOI:** 10.1038/s41598-019-45919-0

**Published:** 2019-07-04

**Authors:** Pingxian Wu, Kai Wang, Qiang Yang, Jie Zhou, Dejuan Chen, Yihui Liu, Jideng Ma, Qianzi Tang, Long Jin, Weihang Xiao, Pinger Lou, Anan Jiang, Yanzhi Jiang, Li Zhu, Mingzhou Li, Xuewei Li, Guoqing Tang

**Affiliations:** 1 Farm Animal Genetic Resources Exploration and Innovation Key Laboratory of Sichuan Province, Sichuan Agricultural University, Chengdu, 611130 Sichuan China; 2 College of Life Science, Sichuan Agricultural University, Yaan, 625014 Sichuan China; 3 Sichuan Animal Husbandry Station, Chengdu, 610041 Sichuan China; 4 Zhejiang Tianpeng Group Co., Ltd., Jiangshan, 324111 Zhejiang China

**Keywords:** Quantitative trait, Genome-wide association studies

## Abstract

Socially affected traits are affected by direct genetic effects (DGE) and social genetic effects (SGE). DGE and SGE of an individual directly quantify the genetic influence of its own phenotypes and the phenotypes of other individuals, respectively. In the current study, a total of 3,276 Large White pigs from different pens were used, and each pen contained 10 piglets. DGE and SGE were estimated for six socially affected traits, and then a GWAS was conducted to identify SNPs associated with DGE and SGE. Based on the whole-genome re-sequencing, 40 Large White pigs were genotyped and 10,501,384 high quality SNPs were retained for single-locus and multi-locus GWAS. For single-locus GWAS, a total of 54 SNPs associated with DGE and 33 SNPs with SGE exceeded the threshold (*P* < 5.00E-07) were detected for six growth traits. Of these, 22 SNPs with pleiotropic effects were shared by DGE and SGE. For multi-locus GWAS, a total of 72 and 110 putative QTNs were detected for DGE and SGE, respectively. Of these, 5 SNPs with pleiotropic effects were shared by DGE and SGE. It is noteworthy that 2 SNPs (SSC8: 16438396 for DGE and SSC17: 9697454 for SGE) were detected in single-locus and multi-locus GWAS. Furthermore, 15 positional candidate genes shared by SGE and DGE were identified because of their roles in behaviour, health and disease. Identification of genetic variants and candidate genes for DGE and SGE for socially affected traits will provide a new insight to understand the genetic architecture of socially affected traits in pigs.

## Introduction

Social interactions between individuals are plentiful, which could contribute to pig’s growth and welfare^1–4^. Social cooperation among pigs (such as group behaviour) would result in positive effects, whereas the competition (such as tail biting) may result in negative effects. Considering the social interaction in the genetic evaluation model, the phenotypic value should be decomposed into a direct effect due to its own characteristic and a social effect originating from others^5^. Both effects can be further decomposed into a heritable component and a non-heritable component. The heritable component of the social effect its called the social genetic effect (SGE)^6,7^. Griffing *et al*. (1967) first reported that the social effects among group individuals distinctly affected the selection processes in a group selection^5^. Amelie Baud *et al*. (2017) reported that the social genetic effect explained up to 29% of phenotypic variance for health and disease traits in mice, that showed the contribution of the social genetic effect was more than the direct genetic effect (explained 21% of phenotypic variance) in some cases^8^. However, the classical quantitative genetic model ignored the SGE among group members in the past several decades. In that case, the genetic variance for the socially affected trait would be incorrectly estimated due to the ignorance of SGE^5,8^.

Previous studies have been demonstrated that the SGE plays an important role in genetic evaluation^4^ and that their genetic parameters for important traits were estimated in pigs^4,5,9^. For average daily gain (ADG), the genetic correlation between direct genetic effect and social effect was low to moderate (0.24) in pigs^10^. The model ignored the pen effects and showed that the heritability of SGE was approximately were about 0.003^11^ and 0.0007^12^ for ADG. The reported social heritabilities were 0.007, 0.001, and 0.003 for body weight, carcass backfat depth, and carcass lean meat content in heavy pigs^13^, respectively. Moreover, Rostellato *et al*. (2015) reported that the social genetic effect contributed largely to total genetic variance for the carcass traits (ranging from 33.2% to 35%) in pigs^13^. Canario *et al*. (2017) showed that the heritability of SGE was 0.001 to 0.004 for pig growth rate and its genetic correlations among direct and SGE were −0.28 to 0.12 in a Swedish pig population^3^.

In general, the pigs were grouped into different pens, and the social effects among members strongly affected pig productive performance. In pigs, the estimation of the social genetic effect was mainly focused on ADG^14^, feed intake, backfat and muscle depth^7^. Several models that considered different fixed factors were built to estimate SGE. The animal model was first extended to SGE in Japanese quail^15^. This model incorporated the competitive effects in classical BLUP (best linear unbiased prediction), and substantially resulted in an extra selection response. Muir^16^ and Bijma *et al*.^17,18^ presented a full model that simultaneously contained both direct genetic effects and social genetic effects. They estimated SGEs for growth traits and found that SGE contributed the main part of genetic variance in growth rate and feed intake in pigs. In summary, the previous studies on SGE mainly focused on the development of an evaluation model and the estimation of genetic parameters. The genetic architecture and causal pathway for SGE are still unknown.

Pig growth traits are affected by direct genetic effects (DGE) and SGE. To investigate the genetic architecture of DGE and SGE, this study conducted a GWAS for DGE and SGE of six growth traits using the whole-genome re-sequenced data from the Large White pig population. The purpose of this study was to estimate the DGE and SGE for six socially affected traits, and reveal the genomic variation and candidate genes for DGE and SGE of six growth traits in pigs.

## Results

### Summary of estimated DGE and SGE

Using this full model **Y** = **Xb** + ***Z***_***D***_***a***_***D***_ + ***Z***_***S***_***a***_***S***_ + ***Wl*** + ***Vg*** + ***e*** (see Materials and Methods), the DGE and SGE were estimated for each socially affected trait in Large White pigs. Table S1 listed the describe statistics about the DGE and SGE for these six traits. The DGE and SGE contained residual effects of 40 genotyped pigs were separately used to conduct GWAS. Variance components were presented in Table 1 for six socially affected traits.

**Table 1:** The variance components (±standard errors) for six socially affected traits in Large White pigs.

Trait	ADG	D100	B100	ADFI	FCR	RFI
${\sigma }_{A}^{2}$	1173.85 ± 142.25	252.56 ± 33.27	131.63 ± 18.96	15311.26 ± 2217.03	42.93 ± 4.56	12476.73 ± 2187.07
${\sigma }_{Adge}^{2}$	1107.79 ± 147.13	212.63 ± 36.36	126.75 ± 27.78	14157.21 ± 3115.23	44.23 ± 8.74	11577.72 ± 2973.81
${\sigma }_{Asge}^{2}$	49.10 ± 9.26	8.36 ± 2.78	4.87 ± 1.56	109.09 ± 43.33	0.08 ± 0.03	97.23 ± 17.98
${\sigma }_{P}^{2}$	5335.67 ± 114.31	631.40 ± 43.26	292.49 ± 17.28	38278.14 ± 1156.69	138.47 ± 9.87	25993.18 ± 894.27
*h* ^2^	0.22 ± 0.01	0.40 ± 0.02	0.45 ± 0.03	0.40 ± 0.02	0.31 ± 0.01	0.48 ± 0.03
${\sigma }_{TBV}^{2}$	5587.34 ± 716.79	103.59 ± 13.31	598.62 ± 48.79	39423.18 ± 3883.17	101.11 ± 8.84	32734.68 ± 3101.25
${\sigma }_{{T}_{P}}^{2}$	27235.32 ± 1173.24	2243.46 ± 178.65	1387.33 ± 91.12	68527.17 ± 2796.35	307.61 ± 4.97	33449.05 ± 1357.95
*T* ^2^	0.22 ± 0.04	0.46 ± 0.06	0.43 ± 0.05	0.58 ± 0.09	0.33 ± 0.04	0.98 ± 0.17
${r}_{{A}_{DS}}$	0.23 ± 0.11	0.58 ± 0.17	0.15 ± 0.09	0.35 ± 0.16	0.23 ± 0.10	0.70 ± 0.23

${\sigma }_{A}^{2}$, additive genetic variance; ${\sigma }_{Adge}^{2}$, genetic variance of direct genetic effects; ${\sigma }_{Asge}^{2}$, genetic variance of social genetic effects; ${\sigma }_{P}^{2}$, phenotypic variance; *h*^2^, classical heritability (${h}^{2}={\sigma }_{A}^{2}/{\sigma }_{P}^{2}$); ${\sigma }_{TBV}^{2}$, total heritable variance; ${\sigma }_{{T}_{P}}^{2}$, total phenotypic variance; T^2^, total heritability (${T}^{2}={\sigma }_{TBV}^{2}/{\sigma }_{{T}_{P}}^{2}$); ${r}_{{A}_{DS}}$, correlation between direct and social genetic effects.

### The single-locus GWAS results using GEMMA

#### For DGE

**Figure 1:**
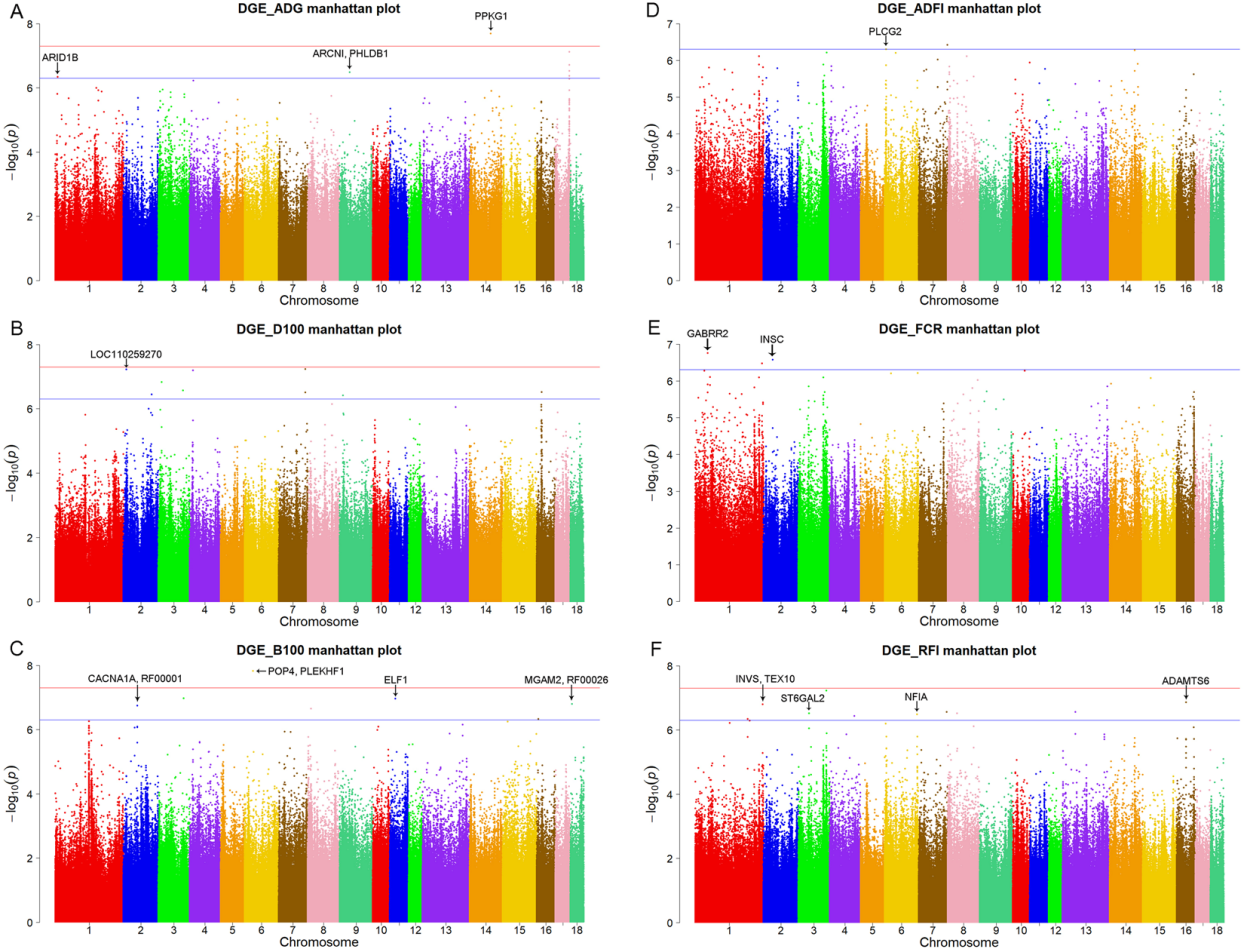
Manhattan plots of genome-wide association analysis results associated to DGE for six growth traits using GEMMA software. The threshold for genome-wide and suggestive significance was set at *P* = 5.00 × 10^−8^ and *P* = 5.00 × 10^−7^, respectively.

A single marker test was performed to identify SNPs associated with DGE of 6 growth traits. The Table 2 and Fig. 1 show the GWAS results for DGE of 6 growth traits. And Q-Q plots are shown in Supplementary Fig. 1. A total of 54 SNPs and 20 genes were detected for DGE in this study.

**Table 2:** The identified SNPs reached the threshold (P < 5.00 × 10^−7^) for DGE of growth traits in Large White pigs.

Trait	Chr	Range of SNP (Mb)	Number of SNP	Position (bp)	n_miss	Allele	MAF	Candidate gene	P-value
dgeADG	1	10.18–10.22	1	10204975	1	G/C	0.308	*ARID1B*	4.50E-07
dgeADG	9	45.89–45.93	4	45909385	1	A/C	0.321	*ARCN1/PHLDB1*	3.29E-07
**dgeADG**	**14**	**98**.**69–98**.**73**	**1**	**98711408**	**0**	**A/G**	**0**.**188**	***PRKG1***	**2**.**05E-08**
dgeADG	17	65.13–65.17	4	65146021	0	A/G	0.325		7.59E-08
dgeD100	2	14.29–14.33	1	14306048	0	G/A	0.450	*LOC110259270*	5.99E-08
dgeD100	2	130.93–130.97	1	130953920	3	C/T	0.149		3.60E-07
dgeD100	3	15.37–15.41	2	15390061	2	G/A	0.276		1.47E-07
dgeD100	3	114.26–114.30	2	114283746	0	C/A	0.350		2.66E-07
dgeD100	4	15.39–15.43	2	15405575	0	C/T	0.450		6.40E-08
dgeD100	7	124.84–124.88	4	124864925	0	A/G	0.250		5.76E-08
dgeD100	9	14.94–10.98	1	14961639	0	A/G	0.138		3.87E-07
dgeD100	16	23.20–23.23	6	23209418	0	C/T	0.100		3.05E-07
dgeB100	2	65.69–65.73	1	65708604	0	T/A	0.150	*CACNA1A/RF00001*	1.78E-07
dgeB100	3	116.98–117.02	1	116995212	4	A/C	0.111		1.04E-07
**dgeB100**	**6**	**39**.**75–39**.**79**	**1**	**39767887**	**0**	**G/A**	**0**.**188**	***POP4/PLEKHF1***	**1**.**49E-08**
dgeB100	8	16.42–16.46	1	16438396	2	G/A	0.474		2.23E-07
dgeB100	11	25.86–25.90	1	25881988	0	C/G	0.375	*ELF1*	1.08E-07
dgeB100	16	7.73–7.77	1	7746260	0	T/C	0.113		4.61E-07
dgeB100	18	7.78–7.82	1	7803229	1	C/T	0.103	*MGAM2/RF00026*	1.59E-07
dgeADFI	6	6.49–6.53	1	6509038	2	T/A	0.145	*PLCG2*	4.98E-07
dgeADFI	7	133.19–133.23	1	133206730	0	T/A	0.275		3.78E-07
dgeFCR	1	57.25–57.29	2	57267261	0	G/C	0.125	*GABRR2*	1.76E-07
dgeFCR	1	309.02–309.06	1	309043978	0	G/C	0.175		3.32E-07
dgeFCR	2	43.99–44.03	1	44010935	0	G/C	0.362	*INSC*	2.62E-07
dgeRFI	1	312.93–312.97	2	312947811	0	T/C	0.350	*INVS/TEX10*	1.57E-07
dgeRFI	1	242.02–242.06	1	242043203	0	C/T	0.125		4.53E-07
dgeRFI	3	48.62–48.66	2	48636349	0	T/C	0.388	*ST6GAL2*	2.99E-07
dgeRFI	3	129.68–129.72	1	129695008	0	C/T	0.263		6.01E-08
dgeRFI	4	114.56–114.60	1	114582555	0	A/T	0.113		3.64E-07
dgeRFI	6	150.80–150.84	1	150817328	0	G/A	0.275	*NFIA*	3.27E-07
dgeRFI	7	129.72–129.76	1	129741749	0	A/G	0.125		2.74E-07
dgeRFI	8	44.17–44.21	1	44189335	0	C/T	0.312		3.00E-07
dgeRFI	13	60.23–60.27	1	60251748	0	C/A	0.163		2.77E-07
dgeRFI	16	43.79–43.83	1	43805766	0	C/T	0.212	*ADAMTS6*	1.38E-07

Chr, Chromosome; Range, Range of significant chromosome region; Number of SNP, Number of SNP involved; Position, Position of top SNP; n_miss, number of missing values of the SNP; Alleles, Alleles of top SNP; MAF, Minor allele frequency; Candidate Gene, Gene found in the range.

At the genome-wide threshold (*P* < 5 × 10^−8^), 1 SNP was associated with dgeADG (Fig. 1A) and 1 with dgeB100 (Fig. 1C). For dgeADG, the genome-wide significant chromosome region was located in SSC14: 98.69–98.73 Mb. The top SNP (SSC14: 98711408 bp, *P* = 2.05 × 10^−8^) was located in the *PRKG1* gene. For dgeB100, the most significant chromosome region was located in SSC6: 39.75–39.79 Mb, and the top SNP (SSC6: 39767887 bp, *P* = 1.49 × 10^−8^) was located in *POP4* and the *PLEKHF1* gene.

**Figure 2:**
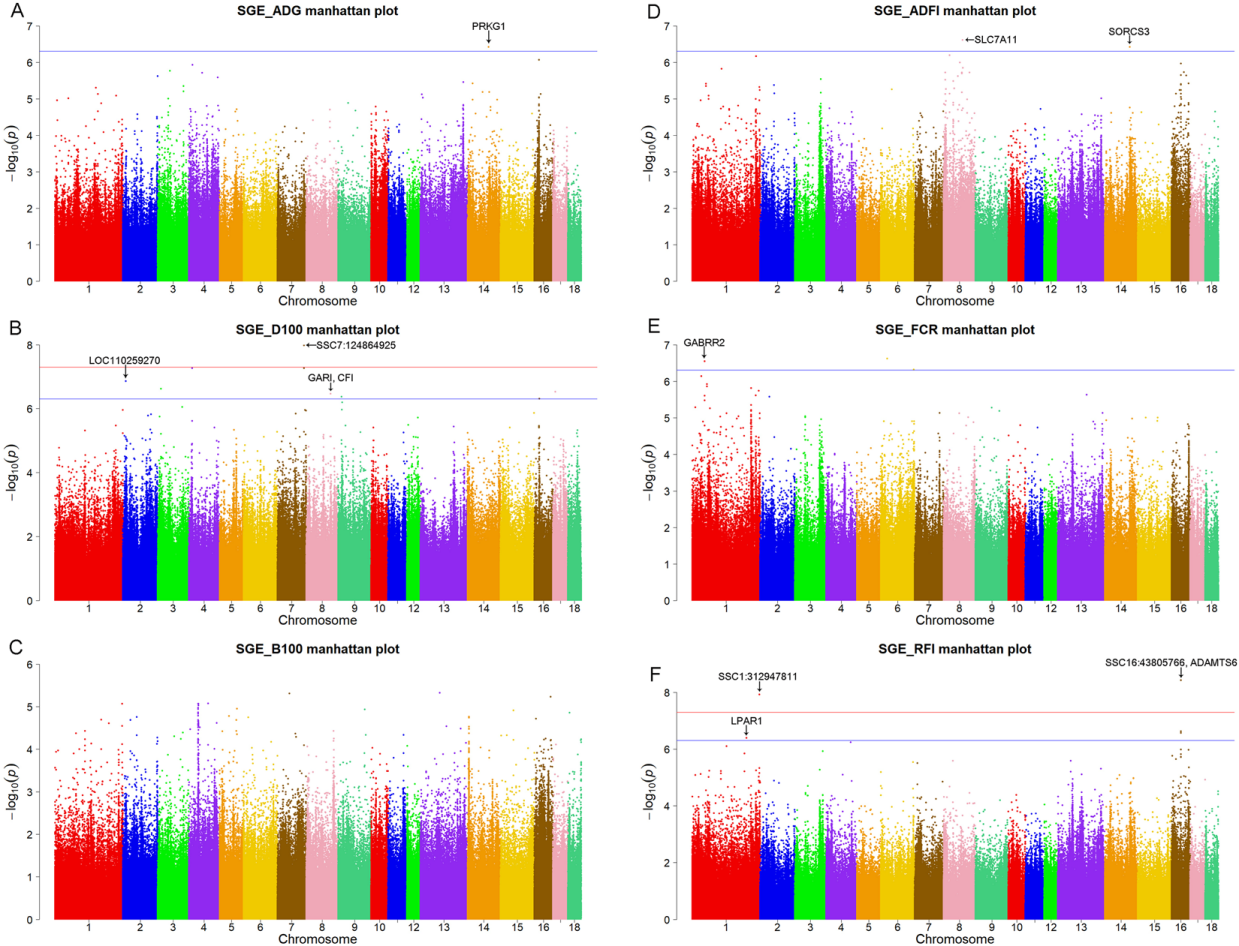
Manhattan plots of genome-wide association analysis results associated to SGE for six growth traits using GEMMA software. The threshold for genome-wide and suggestive significance was set at *P* = 5.00 × 10^−8^ and *P* = 5.00 × 10^−7^, respectively.

At the suggestive threshold (*P* < 5 × 10^−7^), 9 SNPs were associated with dgeADG, 19 with dgeD100, 6 with dgeB100, 2 with dgeADFI, 4 with dgeFCR, and 12 with dgeRFI (Table 2). Of these, 5 top SNPs (including SSC17: 65146021 bp for dgeADG (Fig. 1A); SSC2: 14306048 bp, SSC4: 15405575 bp and SSC7: 124864925 bp for dgeD100 (Fig. 2B); SSC3: 129695008 bp for dgeRFI (Fig. 1F)) were also interesting, since the *P*-value of these SNPs were closed to genome-wide threshold. In the region of SSC2: 14.29–14.33 Mb, the top SNP (SSC2: 14306048 bp, *P* = 5.99 × 10^−8^) was located in *LOC110259270* gene for dgeD100. In addition, a clear peak was observed within the region SS16: 23.20–23.23 Mb for dgeD100, however, no candidate gene was identified in this region.

#### For SGE

To explore the potential genetic variants associated with SGE, a whole-genome re-sequencing association study was conducted for SGE of 6 growth traits. All of the Manhattan plots are shown in Fig. 1 and the Q-Q plots are shown in Supplementary Fig. 2. At the genome-wide threshold (*P* < 5 × 10^−8^), 4 identified SNPs were associated with SGE of days to 100 kg (sgeD100), 7 with sgeRFI. At the lenient threshold (*P* < 5 × 10^−7^) for suggestive associations, 1 SNP was associated with SGE of average daily gain (sgeADG), 14 with sgeD100, 2 with sgeADFI, 4 with sgeFCR, and 1 with sgeRFI, but no association was found for sgeB100 (Table 3 and Fig. 2).

**Table 3:** The identified SNPs reached the threshold (P < 5.00 × 10^−7^) for SGE of growth traits in Large White pigs.

Trait	Chr	Range of SNP (Mb)	Number of SNP	Position (bp)	n_miss	Allele	MAF	Candidate gene	P-value
sgeADG	14	98.69–98.73	1	98711408	0	A/G	0.188	*PRKG1*	3.80E-07
sgeD100	2	14.29–14.33	1	14306048	0	G/A	0.450	*LOC110259270*	1.38E-07
sgeD100	3	15.37–15.41	2	15390061	2	G/A	0.276		2.38E-07
sgeD100	4	15.39–15.43	2	15405575	0	C/T	0.450		5.35E-08
sgeD100	7	124.84–124.88	4	124864925	0	T/C	0.250		1.03E-08
sgeD100	8	112.41–112.45	1	112431517	2	A/G	0.263	*GAR1/CFI*	3.41E-07
sgeD100	9	14.94–14.98	1	14961639	0	A/G	0.138		4.21E-07
sgeD100	16	23.17–23.21	1	23192567	0	A/G	0.100		4.93E-07
sgeD100	16	23.19–23.23	5	23209532	0	G/A	0.100		4.93E-07
sgeD100	17	9.68–9.72	1	9697454	2	G/A	0.434		2.96E-07
sgeADFI	8	88.65–88.69	1	88672493	0	A/G	0.100	*SLC7A11*	2.42E-07
sgeADFI	14	116.10–116.14	1	116122434	0	G/C	0.250	*SORCS3*	3.80E-07
sgeFCR	1	57.25–57.29	2	57267261	0	G/C	0.125	*GABRR2*	2.83E-07
sgeFCR	6	29.60–29.64	1	29619511	0	A/C	0.212		2.38E-07
sgeFCR	6	153.48–153.52	1	153495956	0	T/C	0.287		4.75E-07
sgeRFI	1	312.93–312.97	2	312947811	0	G/A	0.350		1.18E-08
sgeRFI	1	251.97–252.01	1	251994772	0	T/G	0.200	*LPAR1*	3.99E-07
sgeRFI	16	43.79–43.83	5	43805766	0	C/T	0.212	*ADAMTS6*	3.72E-09

Chr, Chromosome; Range, Range of significant chromosome region; Number of SNP, Number of SNP involved; Position, Position of top SNP; n_miss, number of missing values of the SNP; Alleles, Alleles of top SNP; MAF, Minor allele frequency; Candidate Gene, Gene found in the range.

For sgeADG, only one suggestive SNP (*P* = 3.80 × 10^−7^) was distributed on SSC14 (Table 3 and Fig. 2A). One candidate gene (*PRKG1*) was found for sgeADG within the ±20 kb region of the top SNP (SSC14: 98711408 bp).

The results of sgeD100 are shown in Table 3 and Fig. 2B. The most significant locus with 4 consecutive SNPs was identified on SSC7, and the *P*-value of the top SNP (SSC7: 124864925 bp) was 1.03 × 10^−8^. Furthermore, a total of 14 suggestive significant SNPs (*P* < 5 × 10^−7^) for sgeD100 was characterized on SSC2, SSC3, SSC4, SSC8, SSC9, SSC16 and SSC17. On SSC2, the SNP (SSC2: 14306048 bp) was located within the region of the uncharacterized gene (*LOC110259270*). Two genes (*GAR1*, and *CFI*) near the SNP (SSC8: 112431517 bp, *P* = 3.41 × 10^−7^) were found for sgeD100.

A total of 2 suggestive significant SNPs (*P* < 5 × 10^−7^) on SSC8 and SSC14 were detected for sgeADFI (Table 3 and Fig. 2D). In these two significant chromosome regions (SSC8: 88.65–88.69 Mb and SSC14: 116.10–116.14 Mb), two genes (*SLC7A11*, *SORCS3*) were found.

For sgeFCR, a total of 4 associations reached the suggestive significant threshold (*P* < 5 × 10^−7^) on SSC1 and SSC6 (Table 3 and Fig. 2E). In the significant chromosome region (SSC1: 57.25–57.29 Mb), the top SNP (SSC3: 57267261, *P* = 2.83 × 10^−7^) was located in the *GABRR2* gene.

A total of 8 SNPs were found on SSC1 and SSC16 for sgeRFI (Table 3 and Fig. 2F). Two SNPs located on SSC1 and five SNPs located on SSC16 were found to reach the genome-wide significance threshold (*P* < 5 × 10^−8^). The top SNPs (SSC16: 43805766) with the lowest *P*-value of 3.72 × 10^−9^ showed the strongest association effect in the region of 43.79–43.83 Mb. In the significant region of SSC16: 43.79–43.83 Mb, five consecutive SNPs and one promising candidate gene (*ADAMTS6*) were identified for sgeRFI. The second top SNP (SSC1: 312947811) with a low *P*-value of 1.18 × 10^−8^ was found in the region of 312.93–312.97 Mb. In addition, one candidate gene (*LPAR1*) related to disease and healthy were found for sgeRFI in the chromosome regions of SSC1: 251.97–252.01 Mb.

A total of 15 significant SNPs were shared by DGE and SGE that were distributed on SSC1, SSC2, SSC3, SSC4, SSC7, SSC9, SSC14 and SSC16 (Table 4). In the region of SSC14: 98.69–98.73 Mb, the top SNP (SSC14: 98711408 bp) was located in the *PRKG1* gene for dgeADG and sgeADG. In the region of SSC2: 14.29–14.33 Mb, the top SNP (SSC2: 14306048 bp) was located in the *LOC110259270* gene, and in the region of SSC8:112.41–112.45 Mb, the top SNP (SSC8: 112431517 bp) was located in *GAR1* and the *CFI* gene for dgeD100 and sgeD100, respectively.

**Table 4:** The identified SNPs were shared by SGE and DGE of growth traits in Large White pigs.

Trait	Chr	Range of SNP (Mb)	Number of SNP	Position (bp)	Candidate gene	Method
ADG	14	98.69–98.73	1	98711408	*PRKG1*	GEMMA
D100	2	14.29–14.33	1	14306048	*LOC110259270*	GEMMA
D100	3	15.37–15.41	2	15390061		GEMMA
D100	4	15.39–15.43	2	15405575		GEMMA
D100	7	124.84–124.88	4	124864925		GEMMA
D100	9	14.94–14.98	1	14961639		GEMMA
D100	4	131.07–131.11	1	131093320		FarmCPU
D100	8	131.00–131.04	1	131023998		FarmCPU
D100	8	14.25–14.29	1	14272400		FarmCPU
D100	8	80.56–80.60	1	80584807		FarmCPU
D100	8	44.40–44.44	1	44423309		FarmCPU
D100	10	8.96–9.00	1	8978457		FarmCPU
D100	10	11.26–11.30	1	11277917		FarmCPU
FCR	1	57.25–57.29	2	57267261	*GABRR2*	GEMMA
RFI	1	312.93–312.97	1	312947811		GEMMA
RFI	16	43.79–43.83	1	43805766	*ADAMTS6*	GEMMA
ADFI	10	11.00–11.04	1	11015344		FASTmrEMMA
B100	10	11.00–11.04	1	11015344		FASTmrEMMA
D100	4	140.71–140.75	1	140729546		FASTmrEMMA
D100	8	33.10–33.14	1	33116699	*SLC30A9*	FASTmrEMMA
D100	10	11.00–11.04	1	11015344		FASTmrEMMA
D100	11	15.31–15.35	1	15329359	*FOXO1*	FASTmrEMMA
D100	13	217.95–217.99	1	217967861		FASTmrEMMA

Chr, Chromosome; Range, Range of significant chromosome region; Number of SNP, Number of SNP involved; Position, Position of top SNP; Candidate Gene, Gene found in the range.

### The multi-locus GWAS results using FarmCPU

The summary of GWAS results using the FarmCPU approach is listed in Tables S2 and S3. In total, the current study identified 51 and 101 SNPs for DGE and SGE, respectively. For DGE, 4 SNPs were associated with dgeB100, 45 with dgeD100 and 2 with dgeFCR (Table S2), respectively. For SGE, 3 SNPs were associated with sgeADG, 63 with sgeB100, 18 with sgeD100, 7 with sgeADFI, 5 with sgeFCR and 4 with sgeRFI (Table S3), respectively. Among these SNPs, a total of 7 SNPs were shared by dgeD100 and sgeD100 (Table 4). Additionally, three candidate genes were found at 20 kb around these top SNPs for dgeD100 and sgeD100. Among theme, two genes, *NR3C2* and *PKD2*, were separately located in SSC8: 80.56–80.60 Mb and 131.00–131.04 Mb. One gene, *LYPLAL1*, was located in the region of SSC10: 8.96–9.00 Mb.

### The multi-locus GWAS results using FASTmrEMMA

The final results of the FASTmrEMMA method are shown in Tables S4 and S5. For DGE, 16, 18 and 12 putative QTNs were detected for dgeADFI, dgeB100 and dgeD100 (Table S4), respectively. Of these, a QTN SSC8: 16438396 bp located in the region 16.42–16.46 Mb was detected by single-locus GWAS and multi-locus GWAS for dgeB100. This QTN explained 33.19% of the phenotypic variance and included −3.30 QTN effect. For SGE, 19, 15, 14, 5, 15 and 8 putative QTNs were identified for sgeRFI, sgeFCR, sgeD100, sgeB100, sgeADG and sgeADFI (Table S5), respectively. In the region of SSC17: 9.68–9.72 Mb, a QTN located on SSC17: 9697454 bp was identified by single-locus GWAS and multi-locus GWAS for sgeD100. This QTN with 6.04 QTN effect explained 14.30% of the phenotypic variance. Furthermore, a total of 5 putative QTNs were shared by SGE and DGE of each trait (Table 4). For the ADFI, B100 and D100 trait, the QTN SSC10: 11015344 was shared by DGE and SGE. Four QTNs including SSC4: 140729546 bp, SSC8: 33116699 bp, SSC11: 15329359 bp and SSC13: 21796761 bp, were detected in both dgeD100 and sgeD100.

#### Interestingly common loci in the three methods

This study performed a GWAS for DGE and SGE using the GEMMA, FarmCPU and FASTmrEMMA methods. Interestingly, two identified SNPs were validated in three methods (Table 5). One SNP (SSC8: 16438396 bp) was associated with dgeB100. One SNP (SSC17: 9697454 bp) was associated with sgeD100.

**Table 5:** The SNPs validated by three methods.

Trait	Chr	Position	Range of SNP (Mb)	MAF	P-value		
					GEMMA	FarmCPU	FASTmrEMMA
dgeB100	8	16438396	16.42–16.46	0.474	2.96E-07	1.02E-07	1.02E-06
sgeD100	17	9697454	9.68–9.72	0.434	2.23E-07	2.36 E-08	3.50E-07

Chr, Chromosome; Range, Range of significant chromosome region; Position, Position of top SNP; MAF, Minor allele frequency.

## Discussion

### The estimation of genetic effect

Many studies have reported that the social genetic effect are widespread in hens^19^, mice^20^, and pigs^7^. However, at present, very few GWAS for SGE are conducted in pigs because the genetic parameters of SGE are difficult to measure. In past several decades, the heritability of social effects were estimated to be very low in pigs^11,21,22^ using the common REML method. Therefore, social effects were often ignored in past pig breeding. Instead of common heritability of social effects, the ratio between total heritable variance and phenotypic variance was used as a measure of the importance of SGE. On that basis, the social effect was found to contribute the heritable variance in growth rate and feed intake^23^. This study used the full model (including DGE and SGE) described by Bergsma *et al*.^23^ to estimate DGE and SGE of six growth traits. In this model, on one hand, the fixed effects contained sex, tested year and month, the random litter effect and the random group effect were corrected. On the other hand, additional 3236 phenotypic records for ADG, D100, B100, ADFI, FCR and RFI and pedigree information were added into mixed model equations, which improved the prediction accuracy of DGE and SGE for these traits.

### The genetic architecture for SGE

Currently, the genomic information has increased the understanding of complex traits, but the majority of studies have focused only on the DGE of studied traits. Published studies demonstrated that ignoring SGE may severely bias estimates of DGE, and the genetic basis of SGE may be different from the genetic basis of DGE^8^. If SGE contributes to the studied traits, the study of genetic architecture should simultaneously consider DGE and SGE for socially-affected traits. However, few studies have focused on the genetic architecture of SGE. This study simultaneously quantified the SGE and DGE, and performed GWAS to reveal the genetic architecture of DGE and SGE for six growth traits in Large White pigs. Based on the Illumina PorcineSNP60 v2 BeadChip panel, Hong *et al*.^24^ identified several genomic regions and candidate genes associated with SGE for ADG in Landrace pigs. In their study, the individual ADG and the average ADG of unrelated pen-mates were directly used as DGE and SGE, respectively. In our study, the SGE and DGE were estimated for six growth traits, using the full animal model. Then, (1) pseudo phenotype values including DGE and residual effects were used to perform GWAS for DGE of six growth traits; and (2) for SGE of six growth traits, pseudo phenotype values including SGE and residual effects were used to perform a GWAS. Using these pseudo phenotype values, this study revealed the genetic architecture of DGE and SGE for six growth traits in Large White pigs. This study detected 151 and 205 SNPs for DGE and SGE, respectively. These results did not confirm the results with Hong *et al*.^24^. In the present study, the power of QTL detection could be limited by the low estimated heritability of SGE and the small population size in this study. Furthermore the assessment of SGE was different between our study and the previous study^24^. In a previous study, the average ADG of unrelated pen mates was used to detect genetic variants of SGE. However, in our study, the full model **Y** = **Xb** + ***Z***_***D***_***a***_***D***_ + ***Z***_***S***_***a***_***S***_ + ***Wl*** + ***Vg*** + ***e*** (see Materials and Methods) were used to estimate the SGE of each trait, and then the estimated SGE wasused to perform GWAS for SGE of each trait. Our study offered a new insight and effective method for the genetic architecture of socially affected traits in pigs.

### Comparing the single-locus and multi-locus GWAS

To minimize the number of false positives and obtain credible results, the SNPs with MAF > 0.1 were used to perform single-locus and multi-locus GWAS using GEMMA^25^, FarmCPU^26^ and FASTmrEMMA^27,28^ software. Due to the multiple tests, the single-locus GWAS required Bonferroni correction^29^. However, the quantitative traits are controlled by numerous polygenes with large or minor effects, and the Bonferroni correction is overly conservative to identify important loci and may result in false negative results^27^ for complex traits. Recently, some multi-locus GWAS were proposed to detect significant SNPs for complex traits^30,31^. The significant threshold of the multi-locus GWAS is less stringent than single-locus GWAS. Because of the multi-locus and shrinkage natures of this method, a less stringent selection criterion is used in the multi-locus GWAS^31^. Although the multi-locus GWAS used a less stringent criterion, the study used simulated and real data to demonstrated that the multi-locus GWAS using the FASTmrEMMA model is more powerful and has less bias of QTN effect estimation than other methods^31,32^. The FASTmrEMMA method with high power, low false-positive rate and low computing time in QTN detection were used to perform multi-locus GWAS. Using GEMMA, 54 and 33 SNPs reached the threshold of 5.00 × 10^−7^ for DGE and SGE, respectively. Using FarmCPU, 51 and 101 SNPs reached the threshold of 5.00 × 10^−7^ for DGE and SGE, respectively. For FASTmrEMMA, a total of 46 and 71 putative QTNs were detected for DGE and SGE, respectively. Clearly, different methods provided different results. However, all these results show that numerous identified SNPs are associated with pleiotropic effects for multiple socially affected traits. Importantly, two SNPs were detected in three methods. Among them, one SNP (SSC8: 16438396 bp) was located in the region 16.42–16.46 Mb for dgeB100. In this region, four reported QTLs were associated with the B100 trait in pigs^33,34^. One SNP (SSC17: 9697454 bp) was detected in the region of 9.68–9.72 Mb for sgeD100. A previous study reported that numerous QTLs were associated with body weight^35^, behaviour^36^, immune capacity^37^ and disease^38^ in pigs. This SNP may contribute to weight, behaviour, immune and disease traits, and further affect socially affected traits. Thus this study suggested that the SNP SSC17: 9697454 bp was an informative causal locus for SGE in socially affected traits.

### The promising candidate genes identified by GWAS

To investigate the genomic features that contribute to SGE and DGE in pigs, 40 Large White pigs were re-sequenced and 6 socially affected traits were studied. Based on the whole-genome re-sequencing data, this GWAS revealed the genetic architecture for 6 socially affected traits. The previous study only reported a total of 5 QTLs related to socially affected traits in pigs^39^. To date, no genes with their biological functions were reported to associate with SGE in animals. Thus, it is essential to reveal the candidate genes and understand the genetic architecture of DGE and SGE for future pig breeding programmes. In the current study, using a GWAS study, many candidate genes associated with DGE and SGE were found. A total of 20 genes for DGE and 9 genes for SGE were found using single-locus GWAS. A total of 42 and 54 genes were found for DGE and SGE using multi-locus GWAS, respectively. All of these genes were first reported to be associated with SGE in pigs. Additionally, a total of 15 candidate genes associated with pleiotropism for socially affected traits. Among them, 4 genes for single-locus GWAS (including *PRKG1*, uncharacterized *LOC110259270*, *GABRR2* *and ADAMTS6* genes) and 11 genes for multi-locus GWAS (including *SLC26A2*, *HMGXB3*, *SPTB*, *PLEKHG3*, *SLC30A9*, *NRSN2*, *FOXO1, LYPLAL1, NR3C2, PKD2* and *TRIB3* genes) were shared by DGE and SGE, which may imply the pleiotropism of these shared genes for socially affected traits in pigs. These candidate genes were play a role in behaviour, disease and health.

The *PRKG1* gene is a protein coding gene that acts as a key mediator of the cyclic guanosine monophosphate (cGMP) signalling pathway and plays an important role in the cellular signal transduction process. In platelets, hippocampal neurons, smooth muscle and cerebellar Purkinje cells, the *PRKG1* gene was found to be strongly expressed^40,41^. This gene is known to be associated with behaviour^41^, circadian rhythms^42^, sleep^42^, learning^42^ and memory^41^. In humans, the *PRKG1* plays an important role in stress response related traits^43^.

*SORCS3* is an orphan receptor of the VPS10P domain receptor family, a group of sorting and signalling receptors central to many pathways in the control of neuronal viability and function. *SORCS3* plays an important role in the nervous system. This gene was associated with behavioural activities in mice^44^.

On the region of SSC1: 57.25–57.29 Mb, *GABRR2* was found to be associated with dgeFCR and sgeFCR in pigs. *GABRR2* is a protein coding gene that encodes a receptor for gamma-aminobutyric acid (GABA). The GABA receptor regulates the neurotransmitter in the brain^45^, and plays an important role in the behavioral stress response and physiology in animals and humans^45–48^. In mice, the GABA was associated with the aggressiveness and sociability towards conspecifics^47^. The performance and physical condition were affected by GABA in heat-stressed Roman hens^49^.

The strongest association (SSC16: 43805766, *P* = 4.85 × 10^−9^) was located in a disintegrin and metalloproteinase with thrombospondin motifs 6 (*ADAMTS6*). The *ADAMTS6* gene is a member of the *ADAMTS* protein family and is regulated by the cytokine TNF-alpha^50^. The functions of this gene were still not clearly elucidated and mainly reported about aetiology and played a role in the turnover of the extracellular matrix^51^. *ADAMTS6* was expressed differently in each tissue, but the accurate function and the substrates of *ADAMTS6* protein were not clearly demonstrated by a previous study. In past decades, numerous association studies have found the *ADAMTS6* gene to be associated with complex traits in humans. Inguinal hernias^52^ and central corneal thickness and keratoconus^53^ were both identified *ADAMTS6* in a GWAS, which suggested that the *ADAMTS6* gene would affected the collagen homoeostasis in tissues and disorders and lung function. A previous study also suggested that this gene was related to osteosarcoma^54^. Some studies also demonstrated that the *ADAMTS6* gene was significantly upregulated in cancer cells and stromal cells^55^. Importantly, this gene affected the intelligence and growth development, and the balanced translocation disruption of the *ADAMTS6* gene was resulted in short stature and intellectual disability^56^. Given previous studies, *ADAMTS6* gene was associated with growth and disease in biological organisms. The *ADAMTS6* gene was first identified to associate with the sgeRFI. The sgeRFI was not only affected the feed efficiency, but also affected the growth and health of livestock. Considering the pleiotropic effect of this gene, the *ADAMTS6* gene with a potential effect on socially affected traits would be identified as a prominent candidate gene.

In summary, numerous SNPs and genes were identified for DGE and SGE of six traits using single-locus and multi-locus GWAS in Large White pigs. These findings were particularly interesting to better understand the genetic and physiologic mechanisms of both DGE and SGE. Although, a limited number of individuals were used in this study, this study provided a new insight to investigate socially affected traits. Further study using a large population size should contribute to validating the genetic mechanisms for socially affected traits in pigs.

## Materials and Methods

### Ethics statement

All experimental procedures were performed in accordance with the Institutional Review Board (IRB14044) and the Institutional Animal Care and Use Committee of the Sichuan Agricultural University under permit number DKY-B20140302.

### Animals and Trait Recorded

The phenotypic data was collected in 2017, including ADG, days to 100 kg (D100), backfat thickness to 100 kg (B100), average daily feed intake (ADFI), residual feed intake (RFI) and feed conversion ratio (FCR). Initially, the tested pigs were selected between 120 and 130 day of age, with the body weight (BW) about 60 kg. Then a total of 40 Large White pigs from 4 pens were used for this research, containing 27 female and 13 male pigs, and each pen contained 10 piglets homogenous in body weight and age in this study. In this test, these pigs were grouped in the standard commercial pens, which were fed ad libitum by an automatic feeder (The feed intake recording equipment of OSBOREN). The test began at about 65 kg (BW_1_) and the ended at about 110 kg (BW_2_) in the feeding trial.

During the test period, we measured the phenotype data every ten days, and calculated the average value in finally. The ADG, ADFI and FCR were directly calculated from the collected data. The D100 and B100 were calculated as below (Kennedy and Chesnais, unpublished data):

$$\begin{array}{rcl}D100 & = & test\,days-\frac{test\,weight-100}{CF};\\ CF & = & \frac{test\,weight}{test\,days}\times 1.826040\,(male);\\ CF & = & \frac{test\,weight}{test\,days}\times 1.714645\,(female).\\ B100 & = & test\,backfat\,thickness\,(BFT)\times CF;\\ CF & = & \frac{12.402}{12.402+0.106530\times (test\,weight-100)}(male);\\ CF & = & \frac{13.706}{13.706+0.119624\times (test\,weight-100)}(female).\end{array}$$

where CF is the correction coefficient in pig breeding program. Furthermore, the RFI was computed according to the formula (neglecting the lean meat content and dressing percentage)^57^:

$$RFI=ADFI-1.41\,ADG-2.83\,BFT-110.9\,AMW;$$

The average metabolic body weight (AMW) was calculated for each individual using the following classical formula^58^:

$$AMW=(B{W}_{2}^{1.6}-B{W}_{1}^{1.6})/[1.6\times (B{W}_{2}-B{W}_{1})].$$

### Calculate the DGE and SGE

For a socially affected traits, the phenotypic value of an individual can be denoted using following model^5,16^:

$${P}_{i}={A}_{D,i}+{E}_{D,i}+\sum _{j\ne i}^{n-1}\,{A}_{S,j}+\sum _{j\ne i}^{n-1}\,{E}_{S,j},$$

where *P*_*i*_ is the phenotypic value of individual *i*, *A*_*D*,*i*_ is the direct breeding value of individual *i*, *E*_*D*,*i*_ is the direct non-genetic effect of individual *i*, *A*_*S*,*j*_ is the social breeding value of the group member *j*, *E*_*S*,*j*_ is the social non-genetic effect of the group mate *j*, and the *n* is the group size. The *E*_*D*,*i*_ and $\,\sum _{j\ne i}^{n-1}\,{E}_{S,j}$ are combined into a residual component. So,

$${P}_{i}={A}_{D,i}+\sum _{j\ne 1}^{n-1}\,{A}_{S,j}+{E}_{i}.$$

To investigate the direct and social genetic effects, the following full model was built for each trait in this study.

1 $${\bf{Y}}={\bf{Xb}}+{{\boldsymbol{Z}}}_{{\boldsymbol{D}}}{{\boldsymbol{a}}}_{{\boldsymbol{D}}}+{{\boldsymbol{Z}}}_{{\boldsymbol{S}}}{{\boldsymbol{a}}}_{{\boldsymbol{S}}}+{\boldsymbol{Wl}}+{\boldsymbol{Vg}}+{\boldsymbol{e}}$$

where **Y** is the vector of phenotypic observations; **b** is the vector of fixed effects, including sex, tested year and month; ***a***_***D***_ is a vector of DGE; ***a***_***S***_ is a vector of SGE; ***l*** is vector of random litter effects; ***g*** is vector of random group effects; ***e*** is random residual vector; **X**, ***Z***_***D***_, ***Z***_***S***_, ***W*** and ***V*** are incidence matrix of **b**, ***a***_***D***_, ***a***_***S***_, ***l*** and ***g***, respectively. The variance-covariance matrix of ***a***_***D***_ and ***a***_***S***_ is denoted as:

$$\{\begin{array}{cc}{{\boldsymbol{\sigma }}}_{{{\boldsymbol{A}}}_{{\boldsymbol{d}}}}^{2} & {{\boldsymbol{\sigma }}}_{{{\boldsymbol{A}}}_{{\boldsymbol{ds}}}}^{2}\\ {{\boldsymbol{\sigma }}}_{{{\boldsymbol{A}}}_{{\boldsymbol{ds}}}}^{2} & {{\boldsymbol{\sigma }}}_{{{\boldsymbol{A}}}_{{\boldsymbol{s}}}}^{2}\end{array}\}\otimes {\boldsymbol{A}},$$

where ***A*** is additive genetic correlation matrix; ${{\boldsymbol{\sigma }}}_{{{\boldsymbol{A}}}_{{\boldsymbol{d}}}}^{2}$, ${{\boldsymbol{\sigma }}}_{{{\boldsymbol{A}}}_{{\boldsymbol{ds}}}}^{2}$ and ${{\boldsymbol{\sigma }}}_{{{\boldsymbol{A}}}_{{\boldsymbol{s}}}}^{2}$ are genetic variance and covariance between direct effects and social effects. At the *i*^*th*^ row of ***Z***_***S***_, the group members of individual ***i*** were set to 1 and the others were set to 0. For improving the estimation accuracy of DGE and SGE, other 3,236 phenotypic records about ADG, D100, B100, ADFI, FCR and RFI that were obtained from previous performance test also were added into this analysis. These traits were analyzed using the average information restricted maximum likelihood (AI-REML) algorithm by DMU software^59^. Supplementary Table 1 lists the describe statistics about the DGE and SGE for these six traits.

### Genotyping

The pig’s ear tissue was collected from Large White pigs and stored in 75% alcohol. The genomic DNA was extracted by the standard phenol/chloroform method. The Nanodrop-2000 spectrophotometer was used to control the quality of genomic DNA. To obtain nucleotide polymorphism information, a total of 40 Large White pigs were re-sequenced using the Illumina HiSeq PE150 platform, then about 2,000 Gb sequence data was obtained in total. The average sequencing depth of these samples were close to 20×. All the sequence reads were filtered for data quality and mapped to the Sscrofa11.1 reference sequence using BWA software^60^. The mapped reads were realigned by GATK software^61^. A total of 21,104,245 variants were identified using GATK software^61^. In order to minimize the number of false positives in this limited population size, the SNP with MAF > 0.1 were retained for further analysis. Thus, the nucleotide variants were filter based on the quality requirement with minor allele frequency (MAF > 0.1), Missing rate (Miss < 0.1), Hardy-Weinberg equilibrium (HWE < 1.0 × 10^−6^), and read depth (dp > 6) using VCFtools 4.2^62^. Then, the SNPs on the sex chromosome and scaffolds were removed. After the quality control, a total of 10,501,384 SNPs across 18 autosomes were analyzed for association study.

### Single-locus association analysis

The genome-wide efficient mixed-model analysis (GEMMA)^25^ with a univariate liner mixed model were used to perform the single-locus association for DGE and SGE. The analysis models was written as follows:

$${\boldsymbol{y}}={\boldsymbol{Xm}}+{\boldsymbol{Wa}}+{\boldsymbol{e}}$$

where ***y*** is a vector of DGE or SGE contained residual effects from previous model (**1**), ***X*** is the incidence matrix of SNP effects, ***m*** is the vector of SNP effects, ***W*** is the incidence matrix of residual polygene effects, ***a*** is the vector of residual polygene effects, *e* is the random residual vector. Notably, where ***y*** is a vector that phenotype value was corrected by fixed effect, DGE (for SGE), SGE (for DGE), random litter and random group effects.

Recently, several statistical methods have been contributed to determine the significance threshold, such as Bonferroni correction^63^, False discovery rate^64^, and Sidak correction method^65^. In published literatures, the traditional Bonferroni correction method was routinely adopted, but this threshold was not absolute. When identified SNPs exceeded the accepted genome-wide statistical significance threshold *P* < 1.0 × 10^−8^, the GWAS results are most reliable^66,67^. The identified associations (5.00 × 10^−8^ < *P* ≤ 5.00 × 10^−7^) also could be replicated from subsequent studies^28^. The proper significant threshold is various due to different populations, different traits, and different genotypic data^68–70^. Furthermore, the Bonferroni correction is too conservative due to the ignorance of linkages between SNPs, especially for the sequencing data where many adjacent SNPs are highly linked. In the sequencing data, the number of markers used in the Bonferroni correction should be more relevant to the number of segments of chromosome. Thus, the whole-genome significance threshold 5.00 × 10^−8^ was established^71,72^ and the suggestive significant threshold was set at 5.00 × 10^−7^ in this study^73^.

### Multi-locus association analysis

The multi-locus association analysis were performed by FarmCPU^26^ and the fast multi-locus random-SNP-effect EMMA (FASTmrEMMA)^31,74^. FarmCPU iteratively used fixed effect model and random effect model. Using the FASTmrEMMA, this study performed a GWAS for DGE and SGE for six traits in pigs. In this multi-locus GWAS, the random-SNP-effect and multi-locus model methods were used to improve the power and decrease the false-positive rate. At the first stage, the putative quantitative trait nucleotides (QTNs) with *P* ≤ 0.005 were selected for further analysis. Then the selected putative QTNs were analyzed using a multi-locus model for true QTN detection.

### Selecting candidate genes

The bioinformatics databases BioMart (http://www.ensembl.org/) and NCBI (https://www.ncbi.nlm.nih.gov/) were implemented to screen the candidate genes located the significant loci. Only the genes located at the region of ±20 kb around the significant SNPs were considered in this study.

## Supplementary information

Supplementary information

Supplementary Information

## Data Availability

The authors affirm that all of the data necessary for confirming the conclusions made within this article are contained within the article. The dataset used in this study is available from the corresponding author upon reasonable request.
