# Supplementary material for: Whole-genome re-sequencing association study for direct genetic effects and social genetic effects of six growth traits in Large White pigs
Source: Sci Rep. 2019 Jul 4;9:9667. doi: 10.1038/s41598-019-45919-0 (PMC6609718; doi:10.1038/s41598-019-45919-0)
Supplement: Supplementary file 1 — Supplementary information [file 41598_2019_45919_MOESM1_ESM.docx]

**Title Page**

**Article Title**

Whole-genome re-sequencing association study for direct genetic effects and social genetic effects of six growth traits in Large White pigs

**Authors**

Pingxian Wu^1^, Kai Wang^1^, Qiang Yang^1^, Jie Zhou^1^, Dejuan Chen^1^, Yihui Liu^3^, Jideng Ma^1^, Qianzi Tang^1^, Long Jin^1^, Weihang Xiao^1^, Pinger Lou^4^, Anan Jiang^1^, Yanzhi Jiang^2^, Li Zhu^1^, Mingzhou Li^1^, Xuewei Li^1^, Guoqing Tang^1*^

**Authors’ Address**

^1^Farm Animal Genetic Resources Exploration and Innovation Key Laboratory of Sichuan Province, Sichuan Agricultural University, Chengdu 611130, Sichuan, China

^2^College of Life Science, Sichuan Agricultural University, Yaan 625014, Sichuan, China

^3^Sichuan Animal Husbandry Station, Chengdu 610041, Sichuan, China

^4^Zhejiang Tianpeng Group Co., Ltd., Jiangshan 324111, Zhejiang, China

**^*^Corresponding author**

Guoqing Tang, Email: [tyq003@163.com](mailto:tyq003@163.com)

**Co-first author**

Kai Wang, Email: [614351818@qq.com](mailto:614351818@qq.com). This author contributed equally to this work and should be considered co-first authors.

**Authors’ Email**

Pingxian Wu, Email: [wupingxianxian@163.com](mailto:wupingxianxian@163.com)

Kai Wang, Email: [614351818@qq.com](mailto:614351818@qq.com).

Qiang Yang, Email: [836225684@qq.com](mailto:836225684@qq.com).

Jie Zhou, Email: [1048185949@qq.com](mailto:1048185949@qq.com).

Dejuan Chen, Email: [997008941@qq.com](mailto:997008941@qq.com).

Yihui Liu, Email: [YihuiSky@163.com](mailto:YihuiSky@163.com).

Jideng Ma, Email: [jideng_ma@163.com](mailto:jideng_ma@163.com).

Qianzi Tang, Email: [wupie@163.com](mailto:wupie@163.com).

Long Jin, Email: [longjin8806@163.com](mailto:longjin8806@163.com).

Weihang Xiao, Email: [weihang.xiao@sicau.edu.cn](mailto:weihang.xiao@sicau.edu.cn).

Pinger Lou, Email: 435546382@qq.com

Anan Jiang, Email: [30769245@qq.com](mailto:30769245@qq.com).

Yanzhi Jiang, Email: [jiangyz04@163.com](mailto:jiangyz04@163.com).

Li Zhu, Email: [zhuli7508@163.com](mailto:zhuli7508@163.com).

Mingzhou Li, Email: [mingzhou.li@163.com](mailto:mingzhou.li@163.com).

Xuewei Li, Email: [lixuewei9125@126.com](mailto:lixuewei9125@126.com).

Guoqing Tang, Email: [tyq003@163.com](mailto:tyq003@163.com).

Supplementary information


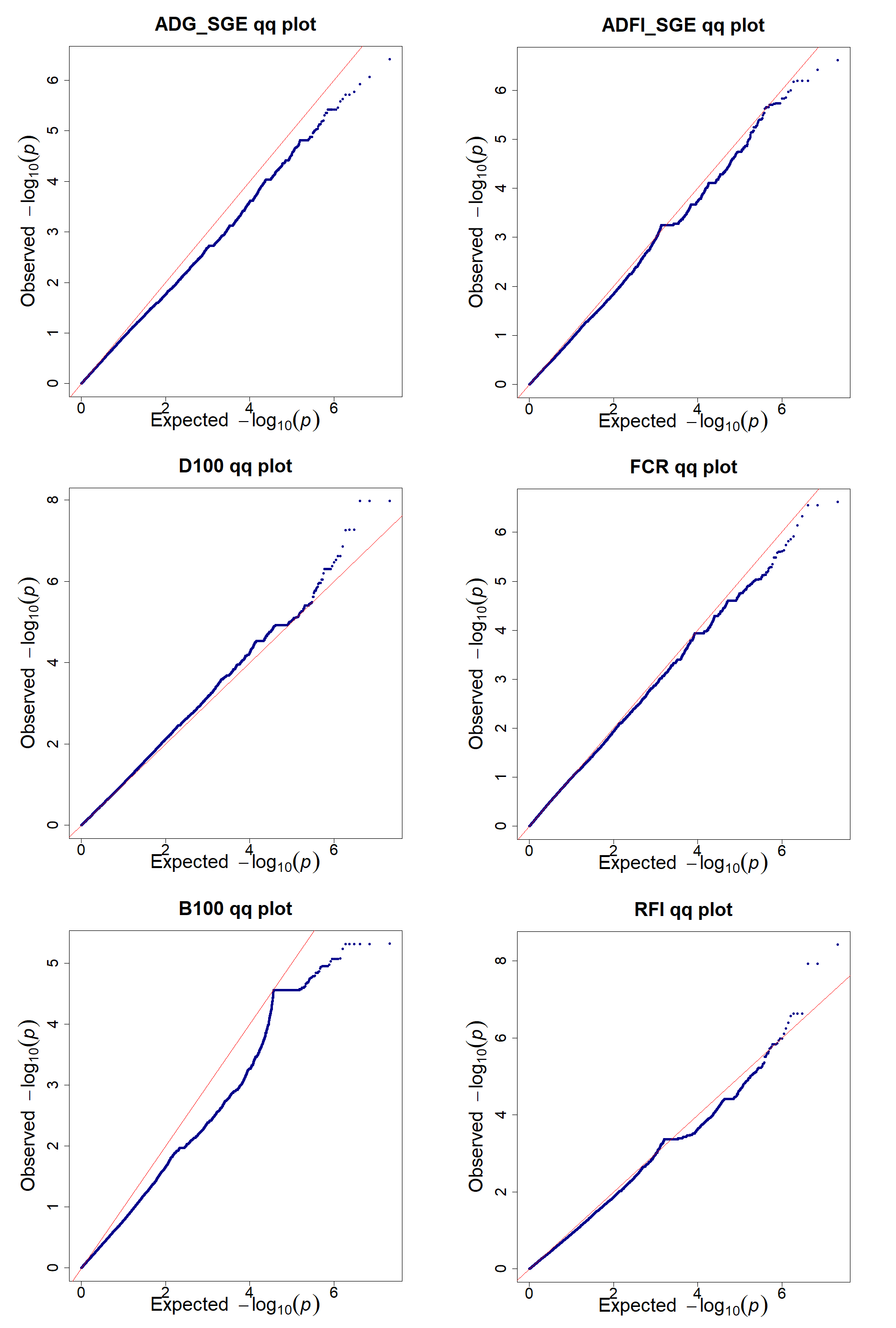


Supplementary Figure 1. The Q-Q plots for SGE of six traits in Large White pigs. The Q-Q plots show the observed −log10-transformed *P*-values (y-axis) and the expected −log10-transformed *P*-values (x-axis).


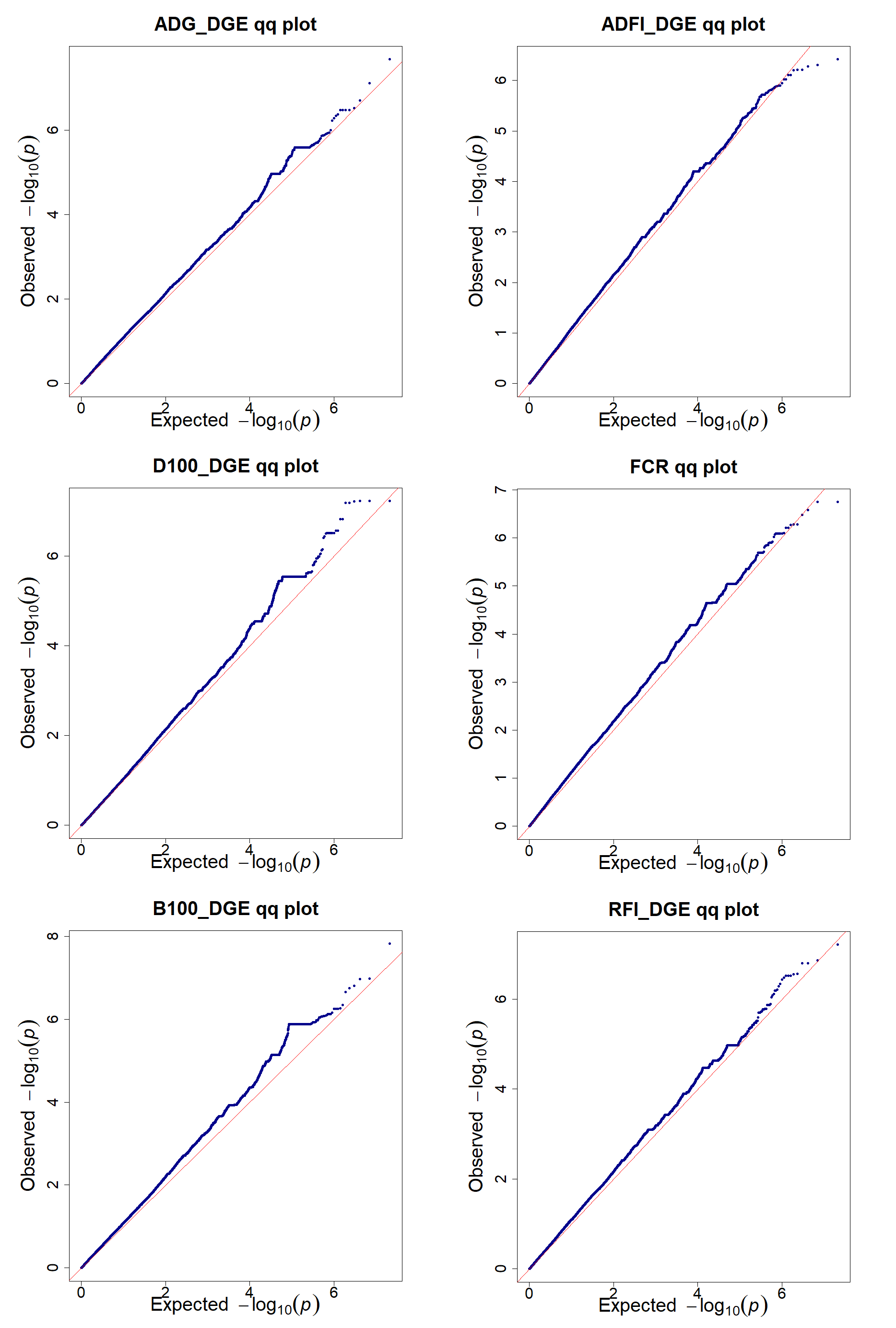


Supplementary Figure 2. The Q-Q plots for DGE of six traits in Large White pigs. The Q-Q plots show the observed −log10-transformed P-values (y-axis) and the expected −log10-transformed P-values (x-axis).

Supplementary Table 1. Descriptive statistics of social and direct genetic effects for six growth traits in Large White pigs.

| Traits | Mean | SD | Min | Max | CV |
| --- | --- | --- | --- | --- | --- |
| dgeADG | -0.16 | 22.02 | -44.51 | 54.18 | -138.17 |
| dgeD100 | -0.7 | 3.05 | -5.04 | 6.33 | -4.36 |
| dgeB100 | 0.2 | 1.82 | -3.33 | 4.73 | 54.35 |
| dgeADFI | -4.47 | 86.17 | -203.12 | 213.91 | -19.26 |
| dgeFCR | -4.00E-03 | 0.06 | -0.14 | 0.1 | -12.94 |
| dgeRFI | -9.88 | 81.73 | -221.27 | 151.8 | -8.27 |
| sgeADG | 0.03 | 1.49 | -3.65 | 3.04 | 46.08 |
| sgeD100 | 0.004 | 0.21 | -0.47 | 0.3 | 54.35 |
| sgeB100 | -0.01 | 0.12 | -0.31 | 0.23 | -23.62 |
| sgeADFI | 0.03 | 5.84 | -14.78 | 13.5 | 186.39 |
| sgeFCR | -5.16E-06 | 3.71E-03 | -7.22E-03 | 8.99E-03 | -7.00E+02 |
| sgeRFI | 0.2 | 5.54 | -10.76 | 14.53 | 27.89 |

Mean, arithmetic mean, SD, standard deviation, Max, maximum; Min, minimum

Supplementary Table 2. The GWAS results of DGE using FarmCPU for six traits in Large White pigs

| Trait | Chr | Range of SNP (Mb) | Number of SNP | Position (bp) | Candidate gene | SNP Effect | P-value |
| --- | --- | --- | --- | --- | --- | --- | --- |
| dgeB100 | 1 | 155.86-155.90 | 1 | 155881793 |  | 1.93 | 9.76E-07 |
|  | 1 | 156.03-156.07 | 2 | 156054435 |  | 1.93 | 9.82E-07 |
|  | **8** | **16.42-16.46** | **1** | **16438396** |  | **3.98** | **1.02E-07** |
| dgeD100 | 13 | 1.59-1.63 | 1 | 1606884 | ATP2C1 | 4.55 | 1.74E-07 |
|  | 4 | 7.00-7.04 | 1 | 7019929 | ZFAT | 2.81 | 2.28E-07 |
|  | 10 | 8.96-9.00 | 1 | 8978457 | LYPLAL1 | 7.97 | 1.10E-18 |
|  | 17 | 9.68-9.72 | 1 | 9697454 |  | -2.52 | 4.10E-07 |
|  | 10 | 11.26-11.30 | 1 | 11277917 |  | 3.50 | 4.85E-07 |
|  | 17 | 12.21-12.25 | 1 | 12225396 |  | -3.61 | 3.00E-08 |
|  | 8 | 14.25-14.29 | 1 | 14272400 |  | 3.19 | 2.30E-07 |
|  | 10 | 17.75-17.79 | 1 | 17769050 | KIF26B | -4.53 | 2.88E-11 |
|  | 1 | 18.05-18.09 | 1 | 18067771 |  | 3.25 | 1.49E-08 |
|  | 3 | 22.21-22.25 | 1 | 22232600 | PRKCB | 3.95 | 4.47E-08 |
|  | 16 | 23.23-23.27 | 1 | 23254267 |  | 13.63 | 2.15E-18 |
|  | 15 | 27.34-27.38 | 1 | 27361225 | CNTNAP5 | -2.79 | 2.32E-07 |
|  | 4 | 31.08-31.12 | 1 | 31103823 | RF00100/OXR1 | 6.13 | 4.85E-15 |
|  | 8 | 33.51-33.55 | 1 | 33525902 | ATP8A1 | 3.39 | 4.50E-07 |
|  | 8 | 44.40-44.44 | 1 | 44423309 |  | 4.48 | 2.43E-07 |
|  | 17 | 49.40-49.44 | 1 | 49415526 |  | 6.09 | 1.16E-11 |
|  | 3 | 51.81-51.85 | 1 | 51830892 | IL1RL1/IL18R1 | -3.49 | 8.63E-07 |
|  | 15 | 52.18-52.22 | 1 | 52195414 |  | 4.44 | 5.23E-10 |
|  | 10 | 52.64-52.68 | 1 | 52663565 |  | 2.85 | 4.56E-07 |
|  | 3 | 55.95-55.99 | 1 | 55970268 | VWA3B | 3.38 | 6.58E-08 |
|  | 17 | 56.13-56.17 | 1 | 56146107 |  | 8.71 | 1.90E-09 |
|  | 11 | 66.12-66.16 | 1 | 66138937 |  | 6.52 | 1.35E-07 |
|  | 10 | 77.78-77.82 | 1 | 77797736 |  | -4.25 | 8.99E-08 |
|  | 11 | 77.90-77.94 | 1 | 77915875 |  | 9.65 | 5.43E-14 |
|  | 16 | 79.60-79.64 | 1 | 79620649 | SLC9A3 | -2.66 | 8.93E-08 |
|  | 16 | 80.50-80.54 | 1 | 80516757 |  | 5.15 | 3.18E-09 |
|  | 8 | 80.56-80.60 | 1 | 80584807 | NR3C2/NR3C2 | 5.54 | 2.49E-08 |
|  | 16 | 81.05-81.09 | 1 | 81070410 |  | 3.97 | 2.29E-07 |
|  | 4 | 96.63-96.67 | 1 | 96650413 | IVL | 3.00 | 8.13E-08 |
|  | 13 | 103.01-103.05 | 1 | 103025360 |  | -2.31 | 4.63E-08 |
|  | 3 | 114.26-114.30 | 2 | 114283746 |  | -8.47 | 1.16E-10 |
|  | 4 | 117.05-117.09 | 1 | 117068231 | S1PR1 | -1.84 | 2.05E-08 |
|  | 4 | 121.91-121.95 | 1 | 121934463 |  | 4.18 | 1.20E-08 |
|  | 8 | 131.00-131.04 | 1 | 131023998 | PKD2 | 7.12 | 9.27E-12 |
|  | 4 | 131.07-131.11 | 1 | 131093320 |  | 7.51 | 3.51E-12 |
|  | 15 | 131.24-131.28 | 1 | 131258825 |  | -4.68 | 1.52E-08 |
|  | 4 | 136.51-136.55 | 1 | 136531970 |  | -3.79 | 2.60E-07 |
|  | 15 | 137.02-137.06 | 1 | 137043598 | COL6A3 | 8.60 | 1.57E-09 |
|  | 1 | 139.47-139.51 | 1 | 139492478 | ALDH1A3/LRRK1 | 14.34 | 2.10E-23 |
|  | 15 | 155.27-155.31 | 1 | 155286999 |  | -7.60 | 1.87E-11 |
|  | 13 | 199.91-199.95 | 1 | 199934594 | DOP1B | 5.55 | 5.82E-13 |
|  | 13 | 203.22-203.26 | 1 | 203244528 | B3GALT5 | 6.80 | 5.02E-11 |
|  | 1 | 295.95-295.99 | 1 | 295972186 |  | -5.18 | 2.05E-10 |
|  | 1 | 304.38-304.42 | 1 | 304396161 |  | 2.42 | 7.03E-08 |
| dgeFCR | 10 | 55.06-55.10 | 2 | 55078806 | MALRD1 | 0.29 | 8.20E-07 |

Chr, Chromosome; Range, Range of significant chromosome region; Position, Position of SNP; Candidate Gene, Gene found in the rang; SNP Effect: the additive effect calculated by FarmCPU

Supplementary Table 3. The GWAS results of SGE using FarmCPU for six traits in Large White pigs

| Trait | Chr | Range of SNP (Mb) | Number of SNP | Position (bp) | Candidate gene | SNP Effect | P-value |
| --- | --- | --- | --- | --- | --- | --- | --- |
| sgeADFI | 15 | 16.26-16.30 | 3 | 16280289 | R3HDM1 | 156.47 | 3.89E-07 |
|  | 9 | 18.78-18.82 | 1 | 18796703 |  | -180.88 | 7.46E-07 |
|  | 12 | 27.93-27.97 | 1 | 27952353 |  | -100.47 | 1.16E-07 |
|  | 2 | 64.31-64.35 | 1 | 64328437 | RF00001/CACNA1A | 136.38 | 9.26E-07 |
|  | 9 | 123.25-123.29 | 1 | 123271401 | CACNA1E | 154.32 | 9.84E-07 |
| sgeADG | 15 | 60.49-60.53 | 1 | 60509307 |  | 47.59 | 2.87E-07 |
|  | 2 | 161.88-161.92 | 1 | 161895925 |  | -40.07 | 4.54E-08 |
|  | 13 | 171.45-171.49 | 1 | 171468948 |  | 30.58 | 6.94E-07 |
| sgeB100 | 12 | 0.68-0.72 | 1 | 699061 | OGFOD3 | 0.42 | 8.70E-08 |
|  | 1 | 7.34-7.38 | 10 | 7363130 | IGF2R | 0.44 | 9.71E-09 |
|  | 5 | 12.57-12.61 | 1 | 12588254 | BPIFC | 0.27 | 4.60E-07 |
|  | 2 | 35.10-35.14 | 1 | 35123442 |  | -0.35 | 2.61E-07 |
|  | 9 | 40.28-40.32 | 1 | 40301996 |  | 0.39 | 4.17E-07 |
|  | 3 | 47.89-47.93 | 1 | 47912968 |  | 0.28 | 6.05E-07 |
|  | 10 | 49.55-49.59 | 1 | 49569339 | MYO3A | 0.22 | 1.24E-08 |
|  | 15 | 60.50-60.54 | 1 | 60517704 |  | 0.31 | 2.54E-07 |
|  | 1 | 63.52-63.56 | 5 | 63535317 |  | 0.24 | 5.06E-08 |
|  | 2 | 65.69-65.73 | 2 | 65708604 |  | 0.33 | 2.09E-07 |
|  | 11 | 71.40-71.44 | 1 | 71422229 |  | 0.30 | 2.99E-07 |
|  | 3 | 71.48-71.52 | 2 | 71496532 | NAGK/PAIP2B | 0.53 | 2.30E-07 |
|  | 1 | 78.22-78.26 | 1 | 78243146 | LAMA4 | 0.44 | 6.84E-07 |
|  | 15 | 94.27-94.31 | 1 | 94288560 | ANKAR | 0.47 | 1.25E-07 |
|  | 1 | 96.90-96.94 | 3 | 96919446 |  | 0.33 | 2.45E-07 |
|  | 3 | 102.38-102.42 | 1 | 102398153 |  | 0.18 | 6.39E-08 |
|  | 3 | 106.49-106.53 | 1 | 106508621 | LTBP1 | -0.27 | 8.47E-08 |
|  | 3 | 107.63-107.67 | 1 | 107647186 |  | -0.28 | 1.50E-07 |
|  | 1 | 113.31-113.35 | 1 | 113329434 | ADAM10 | 0.32 | 8.92E-07 |
|  | 3 | 116.81-116.85 | 1 | 116834882 |  | -0.25 | 9.50E-07 |
|  | 3 | 116.98-117.02 | 1 | 116995212 |  | 0.24 | 2.75E-08 |
|  | 3 | 136.49-136.53 | 1 | 136507358 |  | 0.14 | 1.60E-07 |
|  | 1 | 139.78-139.82 | 4 | 139796774 | CHSY1 | -0.31 | 2.02E-07 |
|  | 1 | 145.86-145.90 | 1 | 145882326 |  | 0.44 | 1.30E-07 |
|  | 15 | 152.74-152.78 | 1 | 152764812 |  | 0.19 | 6.04E-07 |
|  | 2 | 155.72-155.76 | 1 | 155743124 |  | 0.19 | 4.29E-07 |
|  | 2 | 160.07-160.11 | 1 | 160093007 |  | 0.27 | 3.91E-07 |
|  | 2 | 160.98-161.02 | 1 | 160995802 |  | 0.39 | 4.28E-07 |
|  | 1 | 163.77-163.81 | 1 | 163793753 | DENND4A | 0.37 | 1.40E-09 |
|  | 1 | 177.69-177.73 | 1 | 177712827 |  | -0.22 | 6.87E-07 |
|  | 13 | 186.21-186.25 | 3 | 186228413 |  | 0.50 | 1.87E-08 |
|  | 1 | 215.13-215.17 | 2 | 215152378 | KDM4C | -0.30 | 8.08E-08 |
|  | 1 | 250.71-250.75 | 2 | 250730874 | PALM2 | -0.30 | 1.09E-07 |
|  | 1 | 261.69-261.73 | 1 | 261712967 |  | 0.38 | 2.00E-07 |
|  | 1 | 295.60-295.64 | 1 | 295622975 |  | -0.28 | 9.38E-07 |
|  | 1 | 297.02-297.06 | 1 | 297040149 |  | 0.23 | 2.11E-07 |
|  | 1 | 313.02-313.06 | 3 | 313036041 |  | -0.33 | 2.24E-08 |
| sgeD100 | 4 | 1.02-1.06 | 1 | 1041423 | ZC3H3 | 1.67 | 3.28E-08 |
|  | 10 | 8.96-9.00 | 1 | 8978457 | LYPLAL1 | 4.27 | 7.84E-13 |
|  | 10 | 11.26-11.30 | 1 | 11277917 |  | 3.14 | 9.03E-07 |
|  | 8 | 14.25-14.29 | 1 | 14272400 |  | 1.92 | 1.03E-07 |
|  | 4 | 15.38-15.42 | 1 | 15398839 | TMEM65 | 5.37 | 2.67E-12 |
|  | 8 | 16.35-16.39 | 1 | 16374990 |  | -3.72 | 7.99E-08 |
|  | 16 | 21.30-21.34 | 1 | 21319712 | IL7R/CAPSL | 14.14 | 8.92E-16 |
|  | 4 | 36.79-36.83 | 1 | 36808882 | SPAG1 | 2.37 | 1.39E-08 |
|  | 8 | 38.95-38.99 | 1 | 38972178 | DCUN1D4 | -3.70 | 1.11E-07 |
|  | 8 | 44.40-44.44 | 1 | 44423309 |  | 2.85 | 9.53E-07 |
|  | 10 | 53.21-53.25 | 1 | 53230689 | MLLT10 | -2.29 | 1.20E-07 |
|  | 4 | 75.66-75.70 | 1 | 75682400 | PLAG1 | 3.74 | 3.27E-08 |
|  | 8 | 80.56-80.60 | 1 | 80584807 | NR3C2 | 2.74 | 3.45E-07 |
|  | 16 | 85.13-85.17 | 1 | 85146871 |  | 2.33 | 4.07E-07 |
|  | 8 | 131.00-131.04 | 1 | 131023998 | PKD2 | 6.87 | 1.48E-14 |
|  | 4 | 131.07-131.11 | 1 | 131093320 |  | 4.30 | 2.87E-10 |
|  | **17** | **9.68-9.72** | **1** | **9697454** |  | **3.85** | **2.36 E-08** |
|  | 2 | 160.50-160.54 | 1 | 160519138 |  | 0.28 | 9.96E-07 |
| sgeFCR | 15 | 36.50-36.54 | 1 | 36521603 |  | -0.19 | 6.70E-07 |
|  | 18 | 51.64-51.68 | 1 | 51663340 | HECW1 | -0.31 | 1.09E-07 |
|  | 15 | 90.46-90.50 | 1 | 90480040 |  | 0.19 | 8.62E-07 |
|  | 15 | 92.50-92.54 | 2 | 92520660 |  | -0.20 | 6.63E-07 |
| sgeRFI | 6 | 0.00-0.04 | 1 | 23441 |  | 114.31 | 9.81E-07 |
|  | 12 | 27.93-27.97 | 1 | 27952353 |  | -100.47 | 2.63E-07 |
|  | 11 | 74.91-74.95 | 1 | 74926281 |  | -171.47 | 5.36E-07 |
|  | 9 | 123.25-123.29 | 1 | 123271401 |  | 154.32 | 6.63E-07 |
|  | 6 | 150.80-150.84 | 1 | 150817328 | NFIA | -110.61 | 3.91E-07 |

Chr, Chromosome; Range, Range of significant chromosome region; Position, Position of SNP; Candidate Gene, Gene found in the rang; SNP Effect: the additive effect calculated by FarmCPU

Supplementary Table 4. The putative QTNs of multi-locus GWAS (FASTmrEMMA) for DGE of six traits in Large White pigs.

| Trait | Chr | Range of SNP (Mb) | Position (bp) | QTN effect | LOD score | MAF | r^2^ | Candidate genes |
| --- | --- | --- | --- | --- | --- | --- | --- | --- |
| dgeADFI | 2 | 151.02-151.06 | 151041745 | -2.73 | 3.22 | 0.125 | 17.53 | SLC26A2/HMGXB3 |
|  | 3 | 35.37-35.41 | 35385058 | 0.00 | 3.68 | 0.15 | 0.00 |  |
|  | 3 | 102.38-102.42 | 102398153 | -3.03 | 4.50 | 0.2 | 24.99 |  |
|  | 4 | 121.55-121.59 | 121565226 | 0.00 | 3.44 | 0.2 | 0.00 |  |
|  | 7 | 88.78-88.82 | 88800983 | 15.68 | 5.81 | 0.25 | 34.75 | PLEKHG3/SPTB |
|  | 9 | 33.32-33.36 | 33342404 | 15.92 | 7.09 | 0.125 | 33.74 |  |
|  | 10 | 2.88-2.92 | 2904178 | 6.56 | 3.15 | 0.175 | 6.49 |  |
|  | 10 | 11.00-11.04 | 11015344 | 7.68 | 4.78 | 0.225 | 9.93 |  |
|  | 11 | 15.31-15.35 | 15329359 | 9.50 | 6.97 | 0.125 | 11.62 | FOXO1 |
|  | 11 | 26.18-26.22 | 26199799 | 9.46 | 8.88 | 0.175 | 14.33 |  |
|  | 13 | 23.96-24.00 | 23979186 | 0.00 | 3.67 | 0.15 | 0.00 |  |
|  | 15 | 136.61-136.65 | 136625283 | -2.75 | 4.42 | 0.225 | 19.88 |  |
|  | 15 | 147.10-147.14 | 147122053 | -2.48 | 4.36 | 0.25 | 19.38 |  |
|  | 16 | 13.25-13.29 | 13271053 | -196.63 | 3.85 | 0.25 | 15.45 |  |
|  | 16 | 76.49-76.53 | 76512836 | 222.88 | 4.73 | 0.25 | 22.76 |  |
|  | 17 | 34.80-34.84 | 34823297 | 7.54 | 3.18 | 0.25 | 9.04 | TRIB3/NRSN2 |
| dgeB100 | 1 | 276.13-276.17 | 276146056 | -2.11 | 3.24 | 0.175 | 10.35 |  |
|  | 2 | 151.02-151.06 | 151041745 | -2.73 | 3.22 | 0.125 | 17.53 | SLC26A2/HMGXB3 |
|  | 3 | 35.37-35.41 | 35385058 | 0.00 | 3.68 | 0.15 | 0.00 |  |
|  | 3 | 102.38-102.42 | 102398153 | -3.03 | 4.50 | 0.2 | 24.99 |  |
|  | 4 | 121.55-121.59 | 121565226 | 0.00 | 3.44 | 0.2 | 0.00 |  |
|  | 7 | 88.78-88.82 | 88800983 | 15.68 | 5.81 | 0.25 | 34.75 | PLEKHG3/SPTB |
|  | **8** | **16.42-16.46** | **16438396** | **-3.59** | **6.63** | **0.25** | **33.19** |  |
|  | 8 | 19.80-19.84 | 19818352 | -2.55 | 4.97 | 0.225 | 20.17 |  |
|  | 9 | 33.32-33.36 | 33342404 | 15.92 | 7.09 | 0.125 | 33.74 |  |
|  | 10 | 2.88-2.92 | 2904178 | 6.56 | 3.15 | 0.175 | 6.49 |  |
|  | 10 | 11.00-11.04 | 11015344 | 7.68 | 4.78 | 0.225 | 9.93 |  |
|  | 11 | 15.31-15.35 | 15329359 | 9.50 | 6.97 | 0.125 | 11.62 | FOXO1 |
|  | 11 | 26.18-26.22 | 26199799 | 9.46 | 8.88 | 0.175 | 14.33 |  |
|  | 13 | 23.96-24.00 | 23979186 | 0.00 | 3.67 | 0.15 | 0.00 |  |
|  | 15 | 136.61-136.65 | 136625283 | -2.75 | 4.42 | 0.225 | 19.88 |  |
|  | 15 | 147.10-147.14 | 147122053 | -2.48 | 4.36 | 0.25 | 19.38 |  |
|  | 16 | 20.81-20.85 | 20830879 | -2.10 | 3.95 | 0.275 | 16.61 |  |
|  | 17 | 34.80-34.84 | 34823297 | 7.54 | 3.18 | 0.25 | 9.04 | TRIB3/NRSN2 |
| dgeD100 | 3 | 61.54-61.58 | 61557790 | 10.99 | 7.26 | 0.275 | 19.44 |  |
|  | 4 | 140.71-140.75 | 140729546 | 8.44 | 4.24 | 0.25 | 9.59 |  |
|  | 7 | 88.78-88.82 | 88800983 | 15.68 | 5.81 | 0.25 | 34.75 | PLEKHG3/SPTB |
|  | 8 | 33.10-33.14 | 33116699 | 12.27 | 6.32 | 0.125 | 20.17 | SLC30A9 |
|  | 8 | 130.94-130.98 | 130955765 | 10.33 | 5.60 | 0.175 | 16.09 | ABCG2 |
|  | 9 | 33.32-33.36 | 33342404 | 15.92 | 7.09 | 0.125 | 33.74 |  |
|  | 10 | 2.88-2.92 | 2904178 | 6.56 | 3.15 | 0.175 | 6.49 |  |
|  | 10 | 11.00-11.04 | 11015344 | 7.68 | 4.78 | 0.225 | 9.93 |  |
|  | 11 | 15.31-15.35 | 15329359 | 9.50 | 6.97 | 0.125 | 11.62 | FOXO1 |
|  | 11 | 26.18-26.22 | 26199799 | 9.46 | 8.88 | 0.175 | 14.33 |  |
|  | 13 | 217.95-217.99 | 217967861 | 13.10 | 5.86 | 0.25 | 32.35 |  |
|  | 17 | 34.80-34.84 | 34823297 | 7.54 | 3.18 | 0.25 | 9.04 | TRIB3/NRSN2 |

Chr, Chromosome; Range, Range of significant chromosome region; Position, Position of SNP; MAF, Minor allele frequency; r^2^, the proportion of phenotypic variance explained by the putative QTN; Candidate Gene, Gene found in the range

Supplementary Table 5.The putative QTNs of multi-locus GWAS (FASTmrEMMA) for SGE of six traits in Large White pigs.

| Trait | Chr | Range of SNP (Mb) | Position (bp) | QTN effect | LOD score | MAF | r^2^ |  | Candidate gene |
| --- | --- | --- | --- | --- | --- | --- | --- | --- | --- |
| sgeRFI | 2 | 151.02-151.06 | 151041745 | -2.73 | 3.22 | 0.125 | 17.53 |  | SLC26A2/HMGXB3 |
|  | 3 | 35.37-35.41 | 35385058 | 0.00 | 3.68 | 0.15 | 0.00 |  |  |
|  | 3 | 102.38-102.42 | 102398153 | -3.03 | 4.50 | 0.2 | 24.99 |  |  |
|  | 4 | 26.02-26.06 | 26041249 | 95.62 | 3.62 | 0.35 | 21.73 |  |  |
|  | 7 | 88.78-88.82 | 88800983 | 15.68 | 5.81 | 0.25 | 34.75 |  | PLEKHG3/SPTB |
|  | 8 | 2.29-2.33 | 2310013 | 108.58 | 4.80 | 0.175 | 23.11 |  | ADRA2C |
|  | 8 | 16.91-16.95 | 16930675 | 108.39 | 4.05 | 0.175 | 16.73 |  | GBA3 |
|  | 9 | 33.32-33.36 | 33342404 | 15.92 | 7.09 | 0.125 | 33.74 |  |  |
|  | 10 | 2.88-2.92 | 2904178 | 6.56 | 3.15 | 0.175 | 6.49 |  |  |
|  | 10 | 11.00-11.04 | 11015344 | 7.68 | 4.78 | 0.225 | 9.93 |  |  |
|  | 11 | 15.31-15.35 | 15329359 | 9.50 | 6.97 | 0.125 | 11.62 |  | FOXO1 |
|  | 11 | 26.18-26.22 | 26199799 | 9.46 | 8.88 | 0.175 | 14.33 |  |  |
|  | 13 | 23.96-24.00 | 23979186 | 0.00 | 3.67 | 0.15 | 0.00 |  |  |
|  | 15 | 136.61-136.65 | 136625283 | -2.75 | 4.42 | 0.225 | 19.88 |  |  |
|  | 15 | 147.10-147.14 | 147122053 | -2.48 | 4.36 | 0.25 | 19.38 |  |  |
|  | 16 | 12.91-12.95 | 12926042 | -112.56 | 6.75 | 0.25 | 23.55 |  |  |
|  | 16 | 76.49-76.53 | 76512836 | 82.19 | 4.65 | 0.25 | 14.39 |  |  |
|  | 16 | 81.84-81.88 | 81860601 | 101.37 | 4.69 | 0.125 | 14.30 |  |  |
|  | 17 | 34.80-34.84 | 34823297 | 7.54 | 3.18 | 0.25 | 9.04 |  | TRIB3/NRSN2 |
| sgeFCR | 2 | 151.02-151.06 | 151041745 | -2.73 | 3.22 | 0.125 | 17.53 |  | SLC26A2/HMGXB3 |
|  | 3 | 35.37-35.41 | 35385058 | 0.00 | 3.68 | 0.15 | 0.00 |  |  |
|  | 3 | 102.38-102.42 | 102398153 | -3.03 | 4.50 | 0.2 | 24.99 |  |  |
|  | 4 | 26.02-26.06 | 26041249 | 95.62 | 3.62 | 0.35 | 21.73 |  |  |
|  | 6 | 101.25-101.29 | 101273483 | 0.17 | 3.12 | 0.2 | 17.03 |  |  |
|  | 7 | 49.93-49.97 | 49952383 | 0.00 | 3.65 | 0.125 | 0.00 |  | CFAP161 |
|  | 7 | 115.36-115.40 | 115377752 | 0.00 | 8.20 | 0.125 | 0.00 |  | ISG12(A)/IFI27L2 |
|  | 9 | 33.32-33.36 | 33342404 | 15.92 | 7.09 | 0.125 | 33.74 |  |  |
|  | 11 | 15.31-15.35 | 15329359 | 9.50 | 6.97 | 0.125 | 11.62 |  | FOXO1 |
|  | 11 | 26.18-26.22 | 26199799 | 9.46 | 8.88 | 0.175 | 14.33 |  |  |
|  | 13 | 23.96-24.00 | 23979186 | 0.00 | 3.67 | 0.15 | 0.00 |  |  |
|  | 15 | 136.61-136.65 | 136625283 | -2.75 | 4.42 | 0.225 | 19.88 |  |  |
|  | 15 | 147.10-147.14 | 147122053 | -2.48 | 4.36 | 0.25 | 19.38 |  |  |
|  | 16 | 35.87-35.91 | 35890924 | 0.00 | 3.27 | 0.25 | 0.00 |  | MAP3K1 |
|  | 17 | 34.80-34.84 | 34823297 | 7.54 | 3.18 | 0.25 | 9.04 |  | TRIB3/NRSN2 |
| sgeD100 | 1 | 19.84-19.88 | 19860284 | 6.15 | 3.93 | 0.15 | 12.14 |  | FBXO30 |
|  | 4 | 140.71-140.75 | 140729546 | 7.99 | 6.21 | 0.25 | 18.97 |  |  |
|  | 8 | 33.10-33.14 | 33116699 | 5.77 | 5.08 | 0.125 | 9.85 |  | SLC30A9 |
|  | 9 | 132.57-132.61 | 132587663 | 7.30 | 3.79 | 0.125 | 15.31 |  | HHAT |
|  | 10 | 11.00-11.04 | 11015344 | 5.84 | 4.92 | 0.225 | 12.66 |  |  |
|  | 11 | 15.31-15.35 | 15329359 | 6.80 | 5.39 | 0.125 | 13.09 |  | FOXO1 |
|  | 11 | 26.18-26.22 | 26199704 | 6.15 | 5.98 | 0.175 | 13.26 |  |  |
|  | 13 | 217.95-217.99 | 217967861 | 10.03 | 4.77 | 0.25 | 41.77 |  |  |
|  | 14 | 7.65-7.69 | 7670953 | 8.06 | 4.25 | 0.125 | 19.31 |  |  |
|  | 14 | 123.60-123.64 | 123617193 | 5.89 | 4.03 | 0.175 | 12.25 |  |  |
|  | 15 | 20.59-20.63 | 20614739 | 5.34 | 3.19 | 0.225 | 9.79 |  |  |
|  | 16 | 24.44-24.48 | 24464789 | 6.56 | 3.47 | 0.125 | 12.00 |  | FYB1 |
|  | **17** | **9.68-9.72** | **9697454** | **10.24** | **3.76** | **0.275** | **41.03** |  |  |
|  | 18 | 51.82-51.86 | 51835942 | 3.82 | 3.15 | 0.2 | 4.71 |  |  |
| sgeB100 | 8 | 70.77-70.81 | 70788775 | 0.00 | 4.30 | 0.175 | 0.00 |  |  |
|  | 10 | 11.00-11.04 | 11015344 | 5.84 | 4.92 | 0.225 | 12.66 |  |  |
|  | 16 | 65.71-65.75 | 65727168 | 0.00 | 3.09 | 0.125 | 0.00 |  | ADAM19 |
|  | 17 | 9.68-9.72 | 9697454 | 6.04 | 3.76 | 0.275 | 14.30 |  |  |
|  | 18 | 51.82-51.86 | 51835942 | 3.82 | 3.15 | 0.2 | 4.71 |  |  |
| sgeADG | 2 | 151.02-151.06 | 151041745 | -2.73 | 3.22 | 0.125 | 17.53 |  | SLC26A2/HMGXB3 |
|  | 3 | 35.37-35.41 | 35385058 | 0.00 | 3.68 | 0.15 | 0.00 |  |  |
|  | 3 | 102.38-102.42 | 102398153 | -3.03 | 4.50 | 0.2 | 24.99 |  |  |
|  | 4 | 26.02-26.06 | 26041249 | 95.62 | 3.62 | 0.35 | 21.73 |  |  |
|  | 5 | 79.38-79.42 | 79398881 | -39.73 | 3.92 | 0.2 | 24.39 |  | APPL2/WASHC4 |
|  | 6 | 101.25-101.29 | 101273483 | 0.17 | 3.12 | 0.2 | 17.03 |  |  |
|  | 7 | 49.93-49.97 | 49952383 | 0.00 | 3.65 | 0.125 | 0.00 |  | CFAP161 |
|  | 7 | 115.36-115.40 | 115377752 | 0.00 | 8.20 | 0.125 | 0.00 |  | ISG12(A)/IFI27L2 |
|  | 9 | 33.32-33.36 | 33342404 | 15.92 | 7.09 | 0.125 | 33.74 |  |  |
|  | 11 | 15.31-15.35 | 15329359 | 9.50 | 6.97 | 0.125 | 11.62 |  | FOXO1 |
|  | 11 | 26.18-26.22 | 26199799 | 9.46 | 8.88 | 0.175 | 14.33 |  |  |
|  | 13 | 23.96-24.00 | 23979186 | 0.00 | 3.67 | 0.15 | 0.00 |  |  |
|  | 15 | 136.61-136.65 | 136625283 | -2.75 | 4.42 | 0.225 | 19.88 |  |  |
|  | 15 | 147.10-147.14 | 147122053 | -2.48 | 4.36 | 0.25 | 19.38 |  |  |
|  | 17 | 34.80-34.84 | 34823297 | 7.54 | 3.18 | 0.25 | 9.04 |  | TRIB3/NRSN2 |
| sgeADFI | 8 | 70.77-70.81 | 70788775 | 0.00 | 4.30 | 0.175 | 0.00 |  |  |
|  | 10 | 11.00-11.04 | 11015344 | 5.84 | 4.92 | 0.225 | 12.66 |  |  |
|  | 11 | 70.79-70.83 | 70807969 | -176.32 | 6.81 | 0.125 | 34.21 |  |  |
|  | 11 | 77.01-77.05 | 77031435 | 159.60 | 6.20 | 0.2 | 29.14 |  | COL4A2 |
|  | 14 | 104.94-104.98 | 104963460 | -148.05 | 5.74 | 0.2 | 24.12 |  | CEP55 |
|  | 16 | 65.71-65.75 | 65727168 | 0.00 | 3.09 | 0.125 | 0.00 |  | ADAM19 |
|  | 17 | 50.74-50.78 | 50756521 | 147.43 | 3.26 | 0.15 | 18.03 |  | CSE1L |
|  | 18 | 51.82-51.86 | 51835942 | 3.82 | 3.15 | 0.2 | 4.71 |  |  |

Chr, Chromosome; Range, Range of significant chromosome region; Position, Position of SNP; MAF, Minor allele frequency; r^2^, the proportion of phenotypic variance explained by the putative QTN; Candidate Gene, Gene found in the range
